# Serous Papillary Adenofibroma Cyst of the Ovary in a Young Woman: Case Report and Literature Review

**DOI:** 10.3390/life15101601

**Published:** 2025-10-14

**Authors:** Laurențiu Augustus Barbu, Liliana Cercelaru, Valeriu Șurlin, Stelian-Stefaniță Mogoantă, Tiberiu Stefăniță Țenea Cojan, Nicolae-Dragoș Mărgăritescu, Ana-Maria Țenea Cojan, Mihai Popescu, Valentina Căluianu, Gabriel Florin Răzvan Mogoș, Liviu Vasile

**Affiliations:** 1 Department of Surgery, Railway Clinical Hospital Craiova, University of Medicine and Pharmacy of Craiova, 2 Petru Rares Street, 200349 Craiova, Romania; laurentiu.barbu@umfcv.ro (L.A.B.); valentina.andronache@yahoo.com (V.C.); 2 Department of Embryology and Anatomy, University of Medicine and Pharmacy of Craiova, 200349 Craiova, Romania; liliana.cercelaru@umfcv.ro; 3 Department of Surgery, Emergency County Hospital, University of Medicine and Pharmacy of Craiova, 2 Petru Rares Street, 200349 Craiova, Romania; vsurlin@gmail.com (V.Ș.); ssmogo@yahoo.com (S.-S.M.); dmargaritescu@yahoo.com (N.-D.M.); vliviu777@yahoo.com (L.V.); 4 Faculty of Medicine, University of Medicine and Pharmacy of Craiova, 200349 Craiova, Romania; anamariatenea0324@gmail.com; 5 Imaging Department, University of Medicine and Pharmacy of Craiova, 200349 Craiova, Romania; mihai_popescu_rad@yahoo.com

**Keywords:** serous papillary adenofibroma cyst, ovarian benign, tumor adnexal mass, fertility preservation, differential diagnosis, laparoscopy, young women

## Abstract

**Background:** Serous papillary adenofibroma cyst (SPAC) of the ovary is a rare benign epithelial tumor that can mimic borderline or malignant ovarian neoplasms. Reports in young women are particularly scarce. **Purpose:** The aim of this study is to present a rare clinical case of ovarian SPAC in a young woman and to review the existing literature, highlighting diagnostic challenges and implications for fertility-preserving management. **Methods:** We present a clinical case of ovarian SPAC in a 41-year-old woman and conducted a narrative literature review. The search was performed in PubMed, Scopus, and Web of Science to identify reports published between 2000 and 2025. Additional relevant articles were also identified through manual screening of reference lists from selected papers. **Results:** MRI revealed a well-encapsulated septated cystic lesion with solid nodular components and post-contrast enhancement. Tumor markers, including CA 19-9, were elevated. Laparoscopic surgery with intraoperative frozen section confirmed the diagnosis of SPAC, allowing fertility-preserving management. Histopathology established the final diagnosis. **Conclusions:** This case emphasizes the importance of considering SPAC in the differential diagnosis of complex adnexal masses. Early recognition and intraoperative frozen section can guide conservative surgical strategies, avoiding overtreatment and preserving reproductive potential in young patients.

## 1. Introduction

The ovaries are highly vascular endocrine organs responsible for the synthesis of estrogen and progesterone, hormones essential for female reproductive function. The detection of large ovarian cystic tumors, such as giant serous cystadenomas, has become increasingly uncommon in modern practice due to the widespread use of imaging modalities, including ultrasound and computed tomography [1]. Within this context, serous papillary adenofibroma cysts (SPACs) represent a rare benign epithelial neoplasm, with only a limited number of cases reported in the literature [2]. These tumors typically occur in women of reproductive or perimenopausal age, although paraovarian origins have also been described, usually arising from mesonephric or paramesonephric remnants [3].

Although most benign ovarian cystic tumors are epithelial in origin, stromal neoplasms such as fibromas and adenofibromas are also encountered, often incidentally during routine imaging [4]. The diagnosis of SPAC is particularly challenging because its clinical and radiological features frequently overlap with borderline or malignant ovarian tumors. Complex cystic morphology with papillary projections, solid nodules, and occasional elevation of tumor markers (e.g., CA-125, CA 19-9) may mimic malignancy. While ultrasound and MRI are valuable for lesion characterization, neither can reliably exclude borderline or malignant disease. Advances in ultrasound technology, including two-dimensional (2D) imaging with color and power Doppler, permit assessment of vascular patterns within papillary or solid components, offering indirect clues to tumor behavior [5,6]. Three-dimensional (3D) ultrasound further enhances diagnostic accuracy by providing volumetric reconstruction and improved visualization of papillary structures and septations [7]. In settings where MRI is not readily available, transvaginal ultrasound with Doppler evaluation remains the most accessible and rapid modality for preoperative assessment. When combined with validated risk models such as the International Ovarian Tumor Analysis (IOTA) or ADNEX model, and supported intraoperatively by frozen section, clinicians can achieve a reliable diagnosis while avoiding unnecessary radical interventions [8].

Because of these diagnostic uncertainties, patients may undergo overtreatment, including radical surgery in women of reproductive age. Early recognition of SPAC is therefore essential, as intraoperative frozen section can confirm its benign nature and guide fertility-preserving surgical management, minimizing morbidity and safeguarding reproductive potential.

Here, we present a clinical case of ovarian SPAC in a young woman, reviewing its epidemiology, clinical features, and management strategies. This case is notable for the patient’s relatively young age, atypical clinical presentation, and the diagnostic challenges posed by features mimicking malignancy. By documenting this case, we aim to contribute evidence supporting conservative surgical management and to emphasize the importance of including SPAC in the differential diagnosis of complex adnexal masses.

## 2. Literature Review

The majority of ovarian neoplasms arise from the ovarian surface epithelium, a mesothelial layer covering the ovary. These epithelial tumors are classified into subtypes including serous, mucinous, endometrioid, clear cell, ciliated, and Brenner (transitional cell) tumors. Epithelial tumors account for approximately 45% of all ovarian neoplasms, with serous papillary adenomas and adenocarcinomas comprising nearly one-third of this category.

While most benign ovarian tumors originate from surface epithelium, about 25% derive from stromal, sex cord, or paramesonephric (Müllerian) tissues [4]. Tumors with mixed epithelial components, such as serous cystadenomas coexisting with Brenner tumors, suggest a potential Müllerian origin, possibly through metaplastic transformation. By contrast, mesenchymal and germ cell tumors—including fibromas, teratomas, and dysgerminomas—represent distinct entities with separate developmental pathways.

Although benign, these ovarian tumors remain clinically relevant because of their potential to reach large sizes, produce varied clinical presentations, and affect reproductive function. This highlights the importance of surgical management tailored to patient age and fertility considerations.

### 2.1. Epithelial Tumors

Ovarian surface epithelial tumors originate from the mesothelial lining of the ovary or from epithelial remnants within the ovarian hilum. They are classified as benign or malignant and subdivided into five principal histological subtypes: serous, mucinous, endometrioid, clear cell, and transitional (Brenner) tumors [9]. Among benign lesions, serous papillary adenofibroma cysts represent a rare entity, typically presenting as unilateral adnexal masses in women of reproductive or postmenopausal age [9].

Although most benign ovarian tumors arise from surface epithelium, approximately 25% develop from stromal, sex cord, or paramesonephric (Müllerian) tissues [4]. The coexistence of distinct epithelial tumor types, such as serous cystadenomas and Brenner tumors, suggests a possible shared Müllerian origin, potentially through metaplastic transformation. By contrast, mesenchymal and germ cell tumors—including fibromas, teratomas, and dysgerminomas—demonstrate distinct histogenetic pathways [10].

Clinically, serous papillary adenofibroma cysts are often asymptomatic and detected incidentally during imaging or routine gynecologic evaluation. When symptomatic, they typically present due to lesion size or mass effect [11]. The present case adds to the spectrum of rare ovarian and abdominal pathologies that may mimic malignancy. Similarly to mesenteric cysts, which present with nonspecific imaging features and require careful intraoperative evaluation [12,13], SPACs can be misinterpreted as borderline or invasive tumors. Likewise, endometrioid adenofibroma has been described as a rare benign lesion with diagnostic challenges, underscoring the importance of histopathological confirmation [14].

### 2.2. Germ Cell Tumors

Germ cell tumors represent a distinct category of ovarian neoplasms derived from primordial germ cells and include subtypes such as dysgerminomas, teratomas, and choriocarcinomas. Unlike epithelial ovarian tumors, including serous papillary cystadenofibromas, germ cell tumors differ markedly in their clinical presentation, age distribution, and management strategies [9].

Benign epithelial tumors such as serous papillary adenofibroma cysts constitute a minority of ovarian neoplasms. Their clinical manifestations are often nonspecific, usually related to mass effect or incidental detection during imaging. Surgical excision is the standard treatment, with conservative approaches preferred in younger women to preserve fertility when appropriate [15,16,17].

From a clinical perspective, awareness of the histogenetic diversity of ovarian tumors is essential to optimize management decisions and to tailor surgical strategies to both tumor type and individual patient factors [18].

### 2.3. Stromal Tumors

Ovarian stromal tumors, although less frequent than epithelial tumors, represent an important subgroup of ovarian neoplasms. They originate from sex cord–stromal elements and include fibromas, thecomas, granulosa cell tumors, and Sertoli–Leydig cell tumors [19]. While distinct in histogenesis from epithelial tumors, their clinical presentation often overlaps, typically manifesting as abdominal discomfort or pelvic mass effect.

In many cases, stromal tumors are discovered incidentally during imaging performed for unrelated reasons. Symptomatic presentations usually reflect the mass effect rather than aggressive biological behavior. Surgical excision remains the standard of care, with conservative approaches favored when fertility preservation is a priority, particularly in younger patients [20].

Accurate classification of ovarian tumors—whether epithelial, stromal, germ cell, or mixed—is essential for selecting appropriate management strategies and providing reliable prognostic information [21].

### 2.4. Mixed Ovarian Tumors

Mixed ovarian tumors are characterized by the presence of both epithelial and stromal components. This category includes fibromas, composed exclusively of stromal fibroblasts, and adenofibromas, which contain additional epithelial elements, most often of serous or mucinous type [15]. These neoplasms are generally benign, although rare instances of malignant transformation have been reported [19].

Clinically, mixed tumors such as mucinous cystadenomas are frequently associated with Brenner tumors, while associations between serous cystadenomas and Brenner tumors, although less common, have also been described, adding to diagnostic complexity [4].

Management of mixed ovarian tumors follows the same principles as for other benign ovarian neoplasms, with an emphasis on conservative surgical approaches in reproductive-aged women. Careful intraoperative evaluation and individualized surgical planning help to avoid overtreatment, and long-term outcomes are generally favorable [22].

### 2.5. Overview of Serous Papillary Adenofibroma Cyst

Serous papillary adenofibroma cyst (SPAC) is a rare benign ovarian epithelial neoplasm, classified within the group of serous cystic tumors [23]. It typically presents as a unilocular or multilocular ovarian cyst, often detected incidentally during imaging or surgical procedures. Although its pathogenesis is not fully understood, SPAC is thought to originate from the ovarian surface epithelium or epithelial inclusion cysts.

SPAC predominantly affects women of reproductive or perimenopausal age. Clinical manifestations are usually nonspecific, most often pelvic discomfort or abdominal distension. Surgical excision remains the standard treatment, with conservative approaches preferred in younger patients to preserve fertility [23].

#### 2.5.1. Epidemiology and Incidence

Serous papillary adenofibroma cysts (SPACs) are rare benign ovarian tumors that occur most frequently in women between 40 and 65 years of age, although cases in younger patients have also been reported. Their incidence is extremely low within the spectrum of benign ovarian neoplasms, with only a limited number of case reports published to date. Importantly, diagnosis should not be excluded solely on the basis of patient age [24].

#### 2.5.2. Risk Factors

Risk factors for benign ovarian tumors include reproductive age, nulliparity, menorrhagia, and prolonged use of hormonal contraceptives. These tumors are most commonly diagnosed between 35 and 43 years, occasionally after a period of accelerated growth. Large lesions, defined as exceeding 3500 cm^3^ or 500 g, remain relatively uncommon [25].

Hormonal influences, either intrinsic to the tumor or secondary to conditions such as functional cysts or endometriosis, may contribute to tumor enlargement. Acute manifestations, including abdominal pain, ascites, or hemorrhage, may result from complications such as cyst rupture, particularly in large masses. Delayed diagnosis often reflects misinterpretation of abdominal distension or discomfort as obesity, as reported by Kluz et al. [26].

Approximately 75% of benign ovarian tumors are unilateral, while bilateral involvement occurs in about 15%. A positive family history of ovarian or breast cancer is a recognized risk factor [27], whereas long-term oral contraceptive use appears to confer a protective effect [28].

#### 2.5.3. Clinical Presentation and Diagnosis

Serous papillary adenofibroma cysts (SPACs) typically present with vague, non-specific symptoms that often delay diagnosis. Patients may report pelvic discomfort, abdominal distension, urinary frequency, or mild gastrointestinal disturbances, symptoms largely attributable to mass effect [28]. Many patients with small cysts remain asymptomatic, with lesions detected incidentally during routine imaging or gynecologic evaluation [2]. In contrast, large cysts (>10–15 cm) may cause acute complications such as adnexal torsion or rupture [29]. Rare presentations, including ovarian remnant syndrome (ORS) or endocrine manifestations such as irregular menstruation, amenorrhea, or elevated anti-Müllerian hormone (AMH), have been sporadically described, reflecting interference with ovarian hormonal function [25,30].

Transvaginal ultrasound (TVS) remains the first-line diagnostic modality due to its high sensitivity and accessibility. SPAC typically appears as a unilocular or multilocular adnexal mass with thin septa, while Doppler often shows minimal vascularization [29]. However, sonographic findings cannot reliably distinguish SPAC from borderline or malignant tumors when papillary projections or complex internal structures are present [31]. MRI provides superior tissue characterization, with low T2 signal intensity reflecting fibrous stromal content—an important feature that can help differentiate SPAC from malignant tumors [23]. Nevertheless, MRI alone cannot definitively exclude borderline disease, emphasizing the need to integrate imaging with clinical and laboratory data.

Serum tumor markers, particularly CA-125, are commonly assessed preoperatively, but their elevation is nonspecific. Increased CA-125 levels have been reported in benign SPAC, especially in large tumors or in association with peritoneal irritation [1,28]. CA 19-9 may also be elevated, further complicating the differential diagnosis [29]. Therefore, tumor markers must be interpreted cautiously alongside imaging and intraoperative findings.

Intraoperative frozen section (FS) is essential in guiding surgical management. FS provides rapid histopathological evaluation with high specificity (97–100%), allowing fertility-preserving surgery when benignity is confirmed [25]. However, sensitivity may be limited in borderline lesions due to sampling issues [31]. Immunohistochemistry can support diagnosis when FS results are inconclusive, with SPAC typically expressing WT1, ER, PR, and vimentin, and lacking CD10 expression, which helps differentiate it from stromal tumors [2].

In conclusion, accurate diagnosis of SPAC requires a multidisciplinary approach combining clinical assessment, imaging, tumor markers, intraoperative FS, and definitive histopathology. Awareness of its nonspecific presentation and overlapping features with malignancy is essential to prevent overtreatment and to ensure appropriate fertility-preserving surgical management.

#### 2.5.4. Differential Diagnosis

The differential diagnosis of serous papillary adenofibroma cysts (SPACs) primarily includes cystadenofibromas, Brenner tumors, and borderline serous tumors, owing to overlapping histopathological features.

Cystadenofibromas represent the main benign lesions considered. Like SPAC, they exhibit cystic architecture with fibrous stroma, but they more often appear as multilocular cystic masses with extensive papillary projections. Accurate distinction requires careful microscopic evaluation to exclude borderline changes or malignant transformation, particularly at intraoperative frozen section [23].

Brenner tumors may also mimic SPAC due to their dense fibrous stroma. However, they are distinguished by nests of transitional-type epithelial cells. Diagnostic complexity may arise when Brenner tumors coexist with serous cystadenomas or cystadenofibromas, especially if epithelial components are sparse or intermixed [4].

Borderline serous tumors must be excluded when papillary structures or focal epithelial proliferation are present. Unlike SPAC, they demonstrate epithelial stratification, mitotic activity, and nuclear atypia. Since frozen section may not reliably differentiate these entities, permanent section histology remains essential for definitive diagnosis [26].

In rare cases, SPAC itself may contain focal areas suggestive of borderline pathology or malignant potential, underscoring the importance of thorough histopathological evaluation to rule out progression to serous adenofibrocarcinoma [32].

In summary, differentiating SPAC from cystadenofibromas, Brenner tumors, and borderline serous lesions requires integrated assessment of both epithelial and stromal components, supported by intraoperative frozen section and definitive permanent histology.

#### 2.5.5. Management and Prognosis

The management of serous papillary adenofibroma cysts (SPACs) is exclusively surgical, given their benign nature but potential for complications such as torsion or rupture. Conservative techniques are preferred in reproductive-age women to preserve ovarian function. Depending on tumor size, anatomical relationships, and intraoperative findings, options include ovarian cystectomy or unilateral salpingo-oophorectomy [33]. Minimally invasive approaches, particularly laparoscopy, have become the standard when feasible, offering reduced postoperative pain, shorter hospitalization, and faster recovery [23]. However, laparotomy may be required for very large or complex adnexal masses to ensure adequate visualization, prevent rupture, and minimize surgical risks [3]. Radical procedures such as total hysterectomy with bilateral salpingo-oophorectomy are generally reserved for peri- or postmenopausal women or for cases with intraoperative suspicion of borderline or malignant transformation, though this scenario is exceptional in SPAC [27].

Prognosis following complete excision is excellent. Recurrence is extremely rare, and malignant transformation has not been reported to date [30]. Nonetheless, postoperative monitoring is advisable, as rare recurrences or atypical morphological changes may occur. Follow-up should be individualized according to patient age, reproductive goals, tumor size, and complexity. Standard recommendations include a pelvic ultrasound at three months postoperatively, followed by periodic imaging every 6–12 months for the first two years, with longer intervals thereafter if no abnormalities are detected [28]. Although serum CA-125 levels may occasionally be elevated in SPAC, they are nonspecific and not recommended for routine surveillance but may assist in the preoperative differential diagnosis [29]. Overall, individualized follow-up emphasizing imaging and clinical assessment remains the most appropriate strategy, balancing vigilance with the very low risk of recurrence in this benign entity.

### 2.6. Comparative Data from Literature

To provide a broader clinical perspective and illustrate the diagnostic variability of ovarian tumors, Table 1 synthesizes reported cases from the literature, detailing patient age, tumor size, surgical management, histopathological outcomes, and associated intraoperative or biochemical findings. Patient age ranged from the mid-30s to early 60s, with tumor diameters frequently exceeding 10 cm. Most patients underwent total abdominal hysterectomy with bilateral salpingo-oophorectomy (TAH-BSO), often combined with staging procedures or omentectomy in malignant cases. While borderline and malignant epithelial tumors were predominant, benign entities such as fibroma and sex cord–stromal tumors were also described. Common findings included ascites, peritoneal implants, endometriosis, and elevated tumor markers (e.g., CA-125, estrogen), underscoring both the diagnostic challenges and therapeutic complexity of large ovarian masses. Although several authors reported such associated findings, they need to be excluded as they were not observed in our patient. This comparative overview highlights the heterogeneous nature of ovarian tumors and reinforces the need for tailored surgical and diagnostic strategies, particularly in younger patients where fertility preservation remains a central concern.

Serous papillary adenofibroma cysts, though rare and often resembling malignancy, should be considered in the differential diagnosis of adnexal masses, where frozen section can guide safe, fertility-preserving surgery.

### 2.7. Follow-Up Care

Postoperative monitoring is essential following surgical treatment of benign ovarian tumors, with the primary aim of evaluating recovery and detecting potential recurrence. Standard protocols recommend pelvic ultrasonography at three months post-surgery, followed by examinations every six months during the first two years and annually thereafter, with adjustments based on individual clinical factors [28].

Although CA-125 levels may be elevated in serous papillary cystadenofibromas (SPCAF), this marker is nonspecific and may also rise in other benign conditions, potentially causing diagnostic confusion with ovarian carcinoma [26]. Therefore, CA-125 should be used only as an adjunct to imaging and clinical evaluation, not as a primary surveillance tool [28,30].

### 2.8. Long-Term Outcomes

Benign ovarian tumors, including serous papillary adenofibroma cysts (SPACs), are associated with an excellent long-term prognosis after complete surgical excision. Recurrence is exceedingly rare, and malignant transformation has not been documented [30]. Nevertheless, individualized postoperative surveillance remains advisable, tailored to patient age, tumor characteristics, and reproductive goals. Standard follow-up typically consists of periodic clinical assessment and imaging to detect rare recurrences or atypical changes. A personalized approach ensures timely identification of complications while minimizing unnecessary interventions in asymptomatic patients [28].

### 2.9. Genetic Studies

Benign ovarian serous tumors, including serous papillary adenofibroma cysts (SPAC), represent a heterogeneous group of neoplasms predominantly of epithelial origin [23]. Molecular characterization of these lesions remains limited, given their rarity and predominantly benign clinical course. However, early genetic analyses have identified a relatively stable genomic profile, with occasional focal deletions in tumor suppressor genes such as PTEN and ARID1A, suggesting possible involvement of alternative oncogenic pathways in certain cases [30].

Recent transcriptomic studies have noted overexpression of specific proteins, such as PAS domain kinases and monocarboxylate transporter 4, in both benign and non-benign serous tumors, potentially representing future therapeutic targets or diagnostic biomarkers [30]. Nevertheless, routine genetic profiling has not yet been integrated into clinical practice for these benign tumors, given the absence of standardized molecular markers specific to SPAC [38].

Continued molecular research is warranted to clarify tumor pathogenesis and identify biomarkers that may aid in subclassification, prognostic assessment, or therapeutic decision-making [39].

### 2.10. Innovative Treatment Approaches

Ovarian cystadenofibromas, though benign, present therapeutic challenges due to their variable clinical presentation and occasional large size necessitating surgical intervention [4]. The standard approach is surgical excision, with an emphasis on fertility preservation in younger patients and minimally invasive techniques, such as laparoscopy, when feasible [23].

Recent advances focus on optimizing perioperative care and recovery, particularly in patients with large or symptomatic tumors. Enhanced recovery after surgery (ERAS) protocols have been increasingly applied to gynecologic procedures, including ovarian tumor excision, leading to shorter hospital stays and improved outcomes [40].

Although molecularly targeted therapies are not currently indicated for benign ovarian tumors, ongoing genomic and proteomic studies may eventually identify biomarkers or pathways to guide individualized management. At present, treatment remains exclusively surgical, with conservative strategies prioritized to preserve ovarian function and reduce morbidity [4].

### 2.11. Patient Education and Support

Comprehensive patient education is a critical component in the management of ovarian tumors, underscoring the predominantly benign nature of epithelial, germ cell, and sex cord-stromal neoplasms. Educational resources should provide clear information on tumor subtypes, standard treatment approaches, expected outcomes, and potential complications, thereby facilitating informed, shared decision-making [27].

Particular emphasis should be placed on individualized treatment strategies, especially in younger patients where fertility preservation is a priority. Patient materials should address common benign tumors, such as cystadenomas and cystadenofibromas, as well as less frequent entities including teratomas, granulosa cell tumors, and Sertoli–Leydig cell tumors [41]. In addition, the role of surgical management, anticipated recovery, and the importance of long-term follow-up should be highlighted to enhance patient understanding and engagement in care planning [42].

### 2.12. Resources for Patients

Educational programs should aim to clarify the clinical course and management of benign ovarian tumors, including less common entities such as fibromas and cystadenofibromas. Patient-centered resources can reduce anxiety by emphasizing the typically benign nature of these lesions and the favorable outcomes associated with appropriate treatment [27].

Information materials should highlight the importance of individualized surgical management, with particular attention to fertility preservation in younger women. Guidance on postoperative recovery and the need for long-term clinical monitoring further supports patient engagement in care [43].

Awareness initiatives should also stress the value of specialist gynecological assessment, encouraging timely consultations and adherence to recommended follow-up protocols [28].

### 2.13. Support Groups

Advances in molecular profiling have expanded the understanding of ovarian serous neoplasms (OSNs), including rare entities such as serous papillary adenofibroma cysts (SPACs). Genomic analyses demonstrate a largely stable profile with occasional deletions in tumor suppressor genes such as *PTEN* and *ARID1A*, suggesting alternative molecular pathways of tumorigenesis [23].

Emerging research on the expression of PAS domain kinases and monocarboxylate transporter 4 in benign and malignant serous tumors highlights potential novel therapeutic targets [4]. Although currently of theoretical relevance, these molecular markers may eventually assist in distinguishing benign from borderline or malignant lesions during the preoperative assessment.

Clinically, the integration of molecular and genetic data could enhance risk stratification in rare epithelial tumors like SPAC, where imaging and histopathology alone may not provide sufficient guidance for conservative versus radical management. Molecular signatures may also inform individualized monitoring strategies, particularly in younger patients or those with atypical presentations [44].

Future studies correlating molecular alterations with clinical outcomes are needed to refine tumor classification systems and lay the groundwork for personalized management. While histopathology remains the diagnostic cornerstone, the addition of molecular diagnostics has the potential to reduce uncertainty and prevent overtreatment in benign ovarian lesions [45].

### 2.14. Future Directions in Research

Advancements in molecular characterization have enhanced understanding of ovarian serous neoplasms (OSNs), including rare variants such as serous papillary adenofibroma cysts (SPACs). Genomic analyses demonstrate a largely stable genetic profile, with occasional focal deletions in tumor suppressor genes such as *PTEN* and *ARID1A*, suggesting alternative, non-classical pathways of tumorigenesis [23].

Overexpression of PAS domain kinases and monocarboxylate transporter 4, reported in both benign and malignant serous tumors, further points to potential molecular targets with future therapeutic relevance [4]. Although preliminary, these findings highlight the possibility of novel adjunctive markers beyond conventional imaging and histopathology.

Integration of genomic and transcriptomic data into clinical practice could improve subclassification, refine risk stratification, and facilitate earlier recognition of atypical presentations. Precision medicine strategies, while currently applied mainly to malignant ovarian tumors, may eventually be adapted to select benign variants with unusual growth patterns or uncertain biological behavior [46].

Future research should aim to correlate molecular alterations with clinical outcomes in SPAC and other rare benign ovarian tumors. Improved molecular insights may ultimately support individualized monitoring protocols and reinforce conservative surgical management strategies [47].

### 2.15. Potential Therapeutic Targets

Benign ovarian tumors most commonly originate from the ovarian surface epithelium and include serous, mucinous, endometrioid, clear cell, and Brenner subtypes [4]. Within this group, serous papillary adenofibroma cysts (SPACs), a rare variant of serous cystadenoma, usually present as unilateral adnexal lesions identified intraoperatively or incidentally, with bilateral involvement being uncommon [23].

Current evidence suggests that SPAC may arise through transformation of mesothelial or stromal elements, similar to related serous cystic tumors and Brenner tumors [4]. Although hormonal influences have been hypothesized, no definitive molecular or hormonal targets for therapy have yet been established.

At present, management of SPAC remains exclusively surgical, as no systemic treatment options are indicated. Future studies focusing on the epithelial–stromal interface and tumor microenvironment may provide new insights into potential therapeutic pathways. Molecular profiling could eventually refine classification and guide targeted strategies in atypical cases characterized by unusual growth or recurrence [48].

### 2.16. Advancements in Early Detection

Serous papillary adenofibroma cysts (SPACs) are a rare subtype of benign epithelial ovarian tumors, typically smaller than other cystic ovarian lesions [23]. Believed to originate from the ovarian surface epithelium, their molecular pathogenesis remains poorly defined, with no consistent genetic mutations identified to date [27].

Clinically, SPACs are frequently asymptomatic or associated with nonspecific pelvic and gastrointestinal symptoms and are most often detected incidentally during imaging or gynecological evaluation. Although imaging may reveal a complex adnexal mass, conventional radiological assessment lacks the specificity to reliably distinguish SPAC from other benign or malignant epithelial lesions [49].

Current diagnostic approaches focus on improving imaging modalities and incorporating tumor markers to enhance early detection. Nevertheless, management remains primarily surgical, providing both definitive diagnosis and curative treatment [50].

This case illustrates the diagnostic challenges of SPAC, particularly when elevated tumor markers and complex imaging features mimic malignancy. The occurrence in a relatively young woman, coupled with successful fertility-preserving minimally invasive surgery, provides novel clinical insights. These findings underscore the importance of including SPAC in the differential diagnosis of complex adnexal masses to avoid unnecessary radical treatment and to support conservative management when appropriate.

Based on these considerations, we report below a new clinical case of ovarian SPAC in a young woman.

## 3. Case Presentation

### 3.1. Patient Information

A 41-year-old woman with no significant medical history was admitted with a left ovarian cyst, initially detected on pelvic ultrasound and subsequently confirmed by magnetic resonance imaging (MRI). She had previously undergone hormonal therapy without clinical benefit and reported no symptoms such as vaginal discharge or dyspareunia. Biomedical, clinical, and anamnestic characteristics of the patient are summarized in Table 2.

### 3.2. Clinical Findings

On admission, the patient was in stable general condition, with no cardiopulmonary complaints. Abdominal examination revealed localized tenderness in the left lower quadrant, without signs of peritoneal irritation. Rectal examination was unremarkable.

### 3.3. Timeline

A structured timeline of the patient’s presentation, diagnostic workup, surgical intervention, and postoperative course is summarized in Table 3.

### 3.4. Diagnostic Assessment

**Figure 1:**
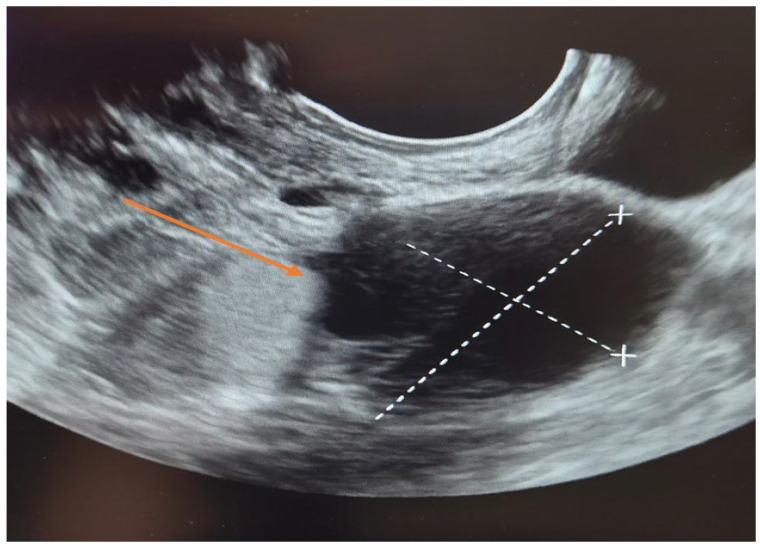
Transvaginal sonography (TVS) of the left adnexa ( orange arrow) demonstrating a complex ovarian cyst with cystic morphology, thin internal septations, and well-defined borders.

The patient was first evaluated using gynecologic ultrasound, including transvaginal sonography (TVS), which is the first-line diagnostic tool for adnexal masses in clinical practice. Transvaginal sonography (TVS) revealed a complex left adnexal mass with cystic morphology, showing well-defined borders and thin internal septations (Figure 1). Subsequently, magnetic resonance imaging (MRI) was performed as a complementary investigation to provide additional characterization of the lesion and to assist in the differential diagnosis with borderline or malignant ovarian tumors.

Laboratory tests revealed mild leukocytosis with elevated inflammatory markers (CRP and fibrinogen), consistent with a nonspecific inflammatory response. These abnormalities were most likely related to the tumor itself and intratumoral hemorrhage, as there was no clinical or imaging evidence of systemic infection. The patient was afebrile and exhibited no peritoneal signs.

Tumor markers were largely within normal limits, except for a modest elevation of CA 19-9. The Risk of Ovarian Malignancy Algorithm (ROMA) score was low (4.5%), indicating a reduced likelihood of ovarian malignancy. Overall, these results supported the suspicion of a benign ovarian lesion rather than a malignant process (Table 4). Human papillomavirus (HPV) genotyping was negative.

**Figure 2:**
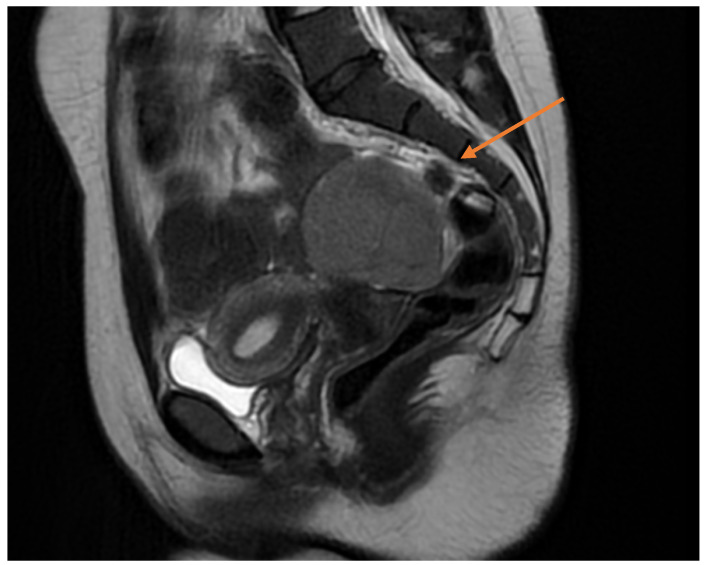
Sagittal pelvic MRI (T2-weighted) demonstrating a septated adnexal mass in the pouch of Douglas, with cystic and nodular components suggestive of intratumoral hemorrhage (yellow arrow).

**Figure 3:**
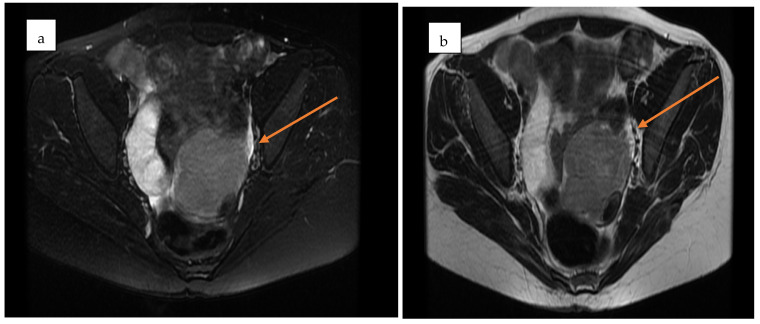
Axial pelvic MRI of the adnexal mass in the pouch of Douglas. (**a**) Pre-contrast T2-weighted image showing a well-encapsulated, septated mass with mixed cystic–solid components (yellow arrow). (**b**) Post-contrast image demonstrating enhancement of the solid nodular component, consistent with vascularization (yellow arrow).

Pelvic MRI demonstrated a well-circumscribed, encapsulated, septated round-oval mass (62 × 74 mm) situated in the pouch of Douglas, adjacent to the left adnexa. The lesion contained multiple small cystic areas and a nodular component with low signal intensity on T2- and T2FS-weighted sequences and high signal intensity on T1, findings consistent with intratumoral hemorrhage (Figure 2 and Figure 3). After contrast administration, the solid component of the mass showed vascular enhancement.

### 3.5. Therapeutic Intervention

**Figure 4:**
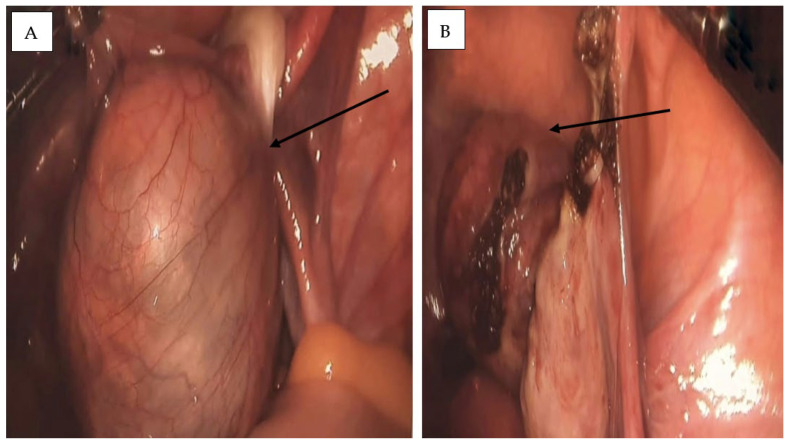
Intraoperative laparoscopic images of the left ovarian cyst. (**A**) A tense, well-defined cystic mass with a smooth surface and prominent vascular markings is observed, partially adherent to the pelvic wall (black arrow). (**B**) Laparoscopic intraoperative view showing the excised ovarian mass within the peritoneal cavity during left adnexectomy. The cyst wall appears friable, with residual adhesions and localized surface changes (black arrow).

The patient underwent laparoscopic surgery under general anesthesia. According to institutional protocol, prophylactic antibiotic prophylaxis was administered perioperatively (single intravenous dose of broad-spectrum antibiotic), as is standard practice in laparoscopic adnexal surgery. Intraoperatively, a **6 × 4 cm left ovarian tumor** was identified, partially adhered to the pelvic wall with mild adhesions. The mass was soft and slightly friable (Figure 4).

A **laparoscopic left adnexectomy** was performed with complete excision of the ovarian cyst. Hemostasis was secured, and no intraoperative complications occurred.

### 3.6. Follow-Up and Outcomes

The postoperative recovery was uneventful. The patient received intravenous antibiotics, tolerated oral intake by postoperative day 1, and was discharged home in good condition on postoperative day 3.

### 3.7. Histopathological Findings

**Figure 5:**
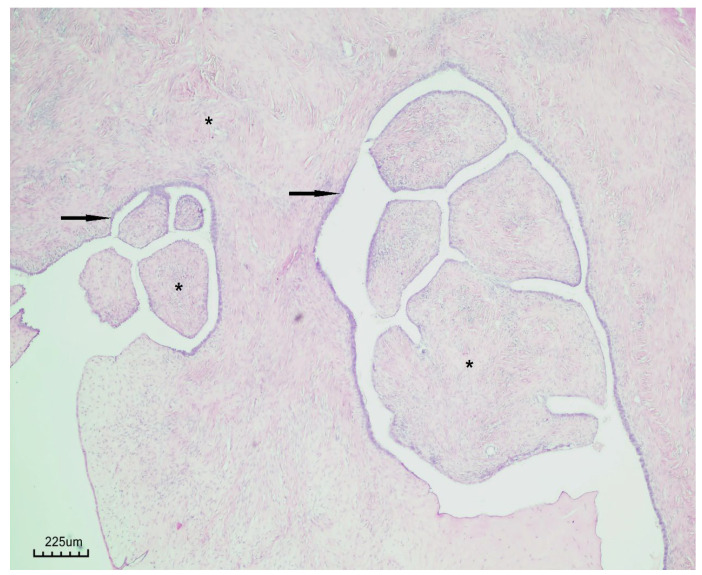
Papillary serous cystadenofibroma (H&E staining, ×40). The cystic wall is lined by simple to pseudostratified epithelium (black arrow), with underlying fibrous stroma (black asterisk).

**Figure 6:**
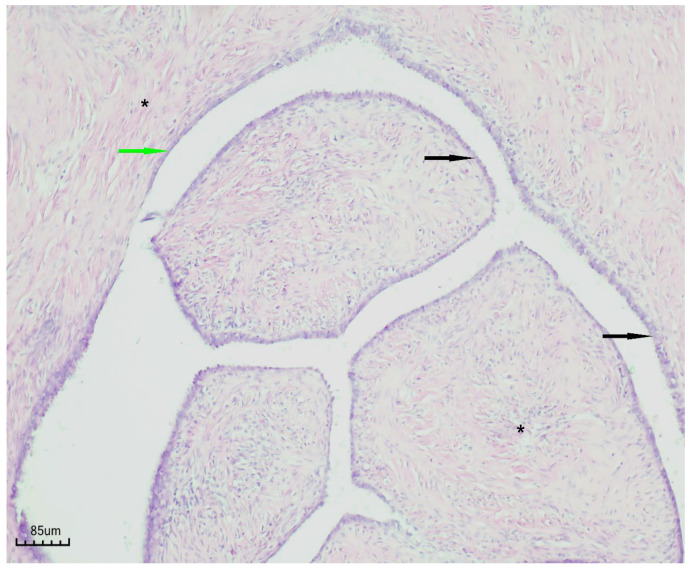
Papillary serous cystadenofibroma (H&E staining, ×100). The cystic wall is indicated by the green arrow, the simple-to-pseudostratified epithelium by the black arrow, and the fibrous stroma by the black asterisk.

Histopathological examination revealed a cystic lesion characterized by branching papillary projections of variable size. The papillae and intervening spaces were lined predominantly by simple to pseudostratified cuboidal or columnar epithelium, with only focal areas of stratification. Ciliated epithelial features were occasionally observed in a focal distribution. The underlying stroma consisted of a proliferation of bland spindle cells arranged mainly in fascicles. Importantly, no evidence of epithelial atypia or stromal invasion was identified (Figure 5 and Figure 6).

## 4. Disscusion

Transvaginal sonography (TVS) is universally regarded as the first-line imaging modality for the evaluation of adnexal masses and remains central to the preoperative diagnosis of serous cystadenofibromas. Virgilio et al. [51] analyzed a large cohort of histologically confirmed cystadenofibromas and highlighted the characteristic sonographic features—typically unilocular solid or multilocular cystic masses with papillary projections, minimal vascularization on Doppler, and frequent acoustic shadowing—features that help distinguish these lesions from malignant tumors. Alcázar et al. [52] similarly described the wide sonographic spectrum of cystadenofibromas and underscored that TVS, particularly in experienced hands, allows recognition of subtle benign indicators that prevent overtreatment. Thus, TVS is critical in the initial diagnostic approach, both for identifying the mass and for guiding surgical decision-making.

Pelvic magnetic resonance imaging (MRI) provides superior tissue characterization and is particularly helpful when TVS findings are inconclusive or when masses extend beyond the pelvis. However, MRI is more expensive, less available, and does not always reliably distinguish borderline from benign tumors. The complementary use of MRI after TVS increases diagnostic accuracy, especially in complex or atypical cases. Therefore, TVS should be emphasized as the cornerstone imaging modality, with MRI serving as a problem-solving tool, while the combined TVS+MRI strategy offers the most robust preoperative characterization when needed.

Our case presents several noteworthy features compared with previously reported cases of serous papillary adenofibroma cyst (SPAC). The patient was relatively young (41 years old, G3P2A1), whereas most reported cases occur in perimenopausal women over 45 years. The hemorrhagic component, rarely described in the literature, added diagnostic complexity, while elevated tumor markers (particularly CA 19-9) created further uncertainty, as such findings are not consistently reported in benign SPAC. Importantly, intraoperative frozen section enabled a fertility-preserving, minimally invasive surgical approach, contrasting with many cases managed by radical surgery.

The hemorrhagic features of this case highlight how bleeding may complicate diagnostic assessment and necessitate careful surgical planning, similar to the impact of comorbidities in gastrointestinal bleeding [15]. By documenting SPAC in a young woman, we underscore both the rarity of this tumor in this age group and the value of conservative surgical management when benignity is confirmed intraoperatively.

Management is primarily surgical, tailored to the patient’s age, reproductive goals, and intraoperative findings. Fertility-preserving approaches are favored in younger women when feasible, with minimally invasive techniques increasingly adopted in suitable cases [16].

A limitation of this report is the absence of Color Doppler/Power Doppler images and Doppler parameters (PS, PI, RI), which were not recorded at the time of the preoperative examination. Nevertheless, the morphological findings on TVS provided sufficient diagnostic information to guide surgical management.

Taken together, the literature emphasizes TVS as the cornerstone of adnexal mass assessment, with MRI serving a complementary role in selected cases. Our case reinforces this complementary role of imaging and highlights the decisive contribution of intraoperative frozen section in establishing benignity. Integrating multimodal imaging with histopathological confirmation maximizes diagnostic confidence and helps avoid unnecessary radical surgery, particularly in younger patients where fertility preservation is a priority.

## 5. Conclusions

Serous papillary adenofibroma cyst (SPAC) is a rare benign ovarian tumor that may mimic borderline or malignant disease, posing significant diagnostic challenges. Our case highlights its occurrence in a relatively young woman, an age group in which fertility preservation is crucial. Imaging and tumor markers alone were insufficient for diagnosis, emphasizing the indispensable role of intraoperative frozen section in guiding safe conservative surgery. By combining case documentation with a literature review, this article contributes novel evidence supporting the inclusion of SPAC in the differential diagnosis of complex adnexal masses and underlines the importance of awareness among gynecologists and radiologists to avoid overtreatment.

## Figures and Tables

**Table 1:** Summary of Reported Ovarian Tumor Cases: Patient Demographics, Tumor Characteristics, Surgical Management, and Histopathological Outcomes.

Study Reference	Age (Years)	Tumor Size (cm)	Diagnostic Tools	Surgical Treatment	Histologic Diagnosis	Other Common Findings
**Bhadoria ** [1]	44	12	CT	TAH-BSO + omentectomy	Advanced serous carcinoma	Pelvic lymphadenopathy
**Agrawal ** [3]	43	13	US + CT	TAH-BSO	Borderline seromucinous tumor	Endometriosis present
**Syam Sundar ** [4]	53	10	NR	TAH-BSO + staging	High-grade serous carcinoma	Hormone receptor negative
**Groutz ** [25]	59	14	US	TAH-BSO + full staging	High-grade serous carcinoma	Ascites present
**Kluz ** [26]	50	10	US; CT/MRI	TAH-BSO	Ovarian fibroma	Solid, firm lesion
**Leelavathi** [23]	52	15	TVS + MRI	TAH-BSO	Borderline mucinous tumor	Multiloculated, mucinous fluid
**Vrabie ** [27]	48	12	N/A	TAH-BSO	Borderline mucinous tumor	Unilateral
**Sivapragasam ** [29]	56	22	NR	TAH-BSO + full staging	Borderline mucinous tumor with carcinoma areas	Peritoneal implants
**Fujita ** [30]	40	9	MRI	Unilateral oophorectomy	Sex cord-stromal tumor (adult granulosa)	Elevated estrogen
**Narayanrao ** [2]	34	17	NR	Unilateral oophorectomy + staging	Benign mucinous tumor with borderline areas	Fertility preservation desired
**Aydin ** [32]	61	16	US + CT	TAH-BSO + omentectomy	Endometrioid carcinoma	Elevated CA-125, peritoneal invasion
**Khatib ** [34]	38	19	CT	Unilateral oophorectomy + biopsies	Borderline mucinous tumor	Moderately elevated CA-125
**Shukla ** [35]	47	11	NR	TAH-BSO	Moderately differentiated endometrioid carcinoma	Bilateral, free peritoneum
**Hunter ** [36]	57	13	N/A	TAH-BSO	Ovarian fibroma	Whitish, solid appearance
**Kumbhar ** [37]	41	14	US	TAH-BSO	Borderline seromucinous tumor	Associated with endometriosis

Legend: TAH-BSO = Total Abdominal Hysterectomy with Bilateral Salpingo-Oophorectomy; CA-125 = Cancer Antigen 125; Staging = limited surgical staging, usually including peritoneal washings and selective peritoneal biopsies; Full staging = comprehensive oncologic staging, typically including peritoneal washings, multiple peritoneal biopsies, omentectomy, and pelvic/paraaortic lymphadenectomy. NR = Not reported; N/A = Not applicable; CT = Computed Tomography; TVS = Transvaginal sonography; US = Ultrasound (type not specified); MRI = Magnetic Resonance Imaging.

**Table 2:** Biomedical, clinical, and anamnestic characteristics of the patient.

Parameter	Value/Findings	Notes
**Age (years)**	41	Young reproductive age
**Weight (kg)**	55	
**Height (cm)**	165	
**BMI (kg/m^2^)**	20.2	Normal weight
**Age at menarche (years)**	15	
**Menstrual history**	Regular cycles	
**Reproductive history**	G3P2A1 (2 full-term deliveries, 1 abortion)	Gravida 3, Para 2, Abortus 1
**Medical history**	No gynecological diseases, no cardiovascular pathology, no other comorbidities reported	Consolidated row
**Family history**	Not reported	
**Hormonal therapy**	Previous treatment without improvement	Mentioned in case
**HPV test**	Negative	

**Table 3:** Clinical Timeline of Patient Presentation and Management.

Day	Clinical Event
**Day 0**	Diagnosis of left ovarian cyst on ultrasound and MRI after 3 months of combined oral contraceptive therapy; no additional MRI was performed between admission and surgery on Day 1.
**Day 1 (Morning)**	Admission to hospital; Clinical examination: abdominal examination revealed localized tenderness in the left lower abdominal quadrant, without peritoneal signs. Rectal examination showed no abnormalities. Gynecological examination revealed a mobile left adnexal mass. Laboratory evaluation included complete blood count (CBC), C-reactive protein (CRP), fibrinogen, coagulation profile, and tumor markers (CA-125, CA 19-9).
**Day 1 (Afternoon)**	Laparoscopic left adnexectomy performed under general anesthesia.
**Postoperative Day 1–2**	Patient received intravenous antibiotics and resumed oral intake.
**Postoperative Day 3**	Discharged home in stable condition without complications.

**Table 4:** Laboratory test results upon admission.

Parameter	Laboratory Results	Normal Range
**White blood cell**	13 × 10^3^ cells/µL	4–10 × 10^3^ cells/µL
**Hemoglobin**	15.2 g/dL	12–16 g/dL
**Platelet count**	210 × 10^3^ cells/µL	150–450 × 10^3^ cells/µL
**Carbohydrate antigen 19.9**	55 U/L	<35 U/L
**Carcinoembryonic antigen**	2 ng/mL	<3 ng/mL
**Carbohydrate antigen 125**	30 U/mL	<35 U/mL
**ROMA score**	4.5%	<7% (premenopausal) *
**Fibrinogen**	450 mg/dL	200–400 mg/dL
**C-reactive protein**	6 mg/dL	<0.5 mg/dL

Legend: * Reference values for ROMA depend on menopausal status; cut-off is <7% in premenopausal and <25% in postmenopausal women.

## Data Availability

The data presented in this study are available on request from the corresponding author. The data are not publicly available due to patient confidentiality.
